# Evaluation of the clinical performance of an AI-based application for the automated analysis of chest X-rays

**DOI:** 10.1038/s41598-023-30521-2

**Published:** 2023-03-05

**Authors:** Julius Henning Niehoff, Jana Kalaitzidis, Jan Robert Kroeger, Denise Schoenbeck, Jan Borggrefe, Arwed Elias Michael

**Affiliations:** Department of Radiology, Neuroradiology and Nuclear Medicine, Johannes Wesling University Hospital, Ruhr University Bochum, Bochum, Germany

**Keywords:** Diseases, Health care, Medical research

## Abstract

The AI-Rad Companion Chest X-ray (AI-Rad, Siemens Healthineers) is an artificial-intelligence based application for the analysis of chest X-rays. The purpose of the present study is to evaluate the performance of the AI-Rad. In total, 499 radiographs were retrospectively included. Radiographs were independently evaluated by radiologists and the AI-Rad. Findings indicated by the AI-Rad and findings described in the written report (WR) were compared to the findings of a ground truth reading (consensus decision of two radiologists after assessing additional radiographs and CT scans). The AI-Rad can offer superior sensitivity for the detection of lung lesions (0.83 versus 0.52), consolidations (0.88 versus 0.78) and atelectasis (0.54 versus 0.43) compared to the WR. However, the superior sensitivity is accompanied by higher false-detection-rates. The sensitivity of the AI-Rad for the detection of pleural effusions is lower compared to the WR (0.74 versus 0.88). The negative-predictive-values (NPV) of the AI-Rad for the detection of all pre-defined findings are on a high level and comparable to the WR. The seemingly advantageous high sensitivity of the AI-Rad is partially offset by the disadvantage of a high false-detection-rate. At the current stage of development, therefore, the high NPVs may be the greatest benefit of the AI-Rad giving radiologists the possibility to re-insure their own negative search for pathologies and thus boosting their confidence in their reports.

## Introduction

In recent years, the corona pandemic has once again shown that medical staff are exposed to an extremely high level of stress in their clinical routine^1,2^. The use of artificial intelligence (AI) in medical care has been discussed extensively for several years in order to support medical staff with the increasing workload in their daily routine—especially in highly technical fields such as radiological departments that deal with image-based tasks^3,4^.

Although various AI-based applications are principally conceivable in medicine, the evaluation of chest radiographs appears to be a good opportunity to establish an AI-based algorithm in clinical routine^5^. Eltorai et al. conducted an online survey in which they asked both radiologists and computer science experts about their expectations regarding the future impact of AI applications on the field of radiology. As part of this survey, they also asked radiologists about their desire for specific AI applications. About 30% of the radiologists declared an interest in AI applications that detect atelectasis (29.5%), pleural effusions (30.5%) and consolidations (31.6%). Even more radiologists expressed their interest in AI applications that indicate pneumothoraces (56.8%) and pulmonary nodules (88.4%)^6^.

The majority of studies evaluating the performance of AI-based algorithms for the interpretation of chest radiographs focus on one particular finding, e.g. signs of COVID-19 infection or tuberculosis^7–14^. The AI-based detection of lung nodules has also been aim of various studies in the past^15–17^.

Siemens Healthineers (Erlangen, Germany) offers an AI-based application for the automated analysis of radiographs of the chest, which continuously aims to develop a holistic approach to patient care. Currently, the AI Rad Companion Chest X-ray (AI-Rad) is designed to detect five specific radiographic findings: pulmonary lesions, consolidation, atelectasis, pneumothorax and pleural effusion. The AI-Rad is considered a diagnostic aid to support radiologists in their clinical routine.

Homayounieh et al. have tested the AI-Rad algorithm with regard to the detection of lung nodules^15^. Their study included 100 p.a. chest radiographs that were evaluated by nine radiologists with different levels of experience. Each radiologist reviewed all images in two sessions—once in an unaided mode, once in AI-aided mode. In the AI-aided session, the mean sensitivity, specificity and detection accuracy for the detection of lung nodules among all radiologists improved by 10.4%, 2.4% and 6.4% compared to unaided session. Junior radiologists experienced greater improvements in sensitivity compared to senior radiologists, whereas all radiologists experienced similar improvements in specificity^15^.

The purpose of the present study is to evaluate the performance of the AI-Rad. We compared the performance metrics of the AI-Rad with those of clinical radiologists by analyzing the findings described in the written reports and the findings detected by the AI algorithm.

## Methods

### Patient population

All radiographs were performed for diagnostic reasons. In total, 499 consecutive patients, who were examined between August and September 2021, were retrospectively enrolled in this study. Patients were not preselected regarding any personal characteristics (e.g. weight, age, gender) or certain pathologies. The radiographs were acquired with seven different X-ray devices that are located in four different hospitals. All hospitals are part of our radiological department.

### AI rad companion chest X-ray

The AI-Rad solely analyzes the posterior-anterior (p.a.) view of chest X-ray images and creates secondary capture DICOM objects reporting on the results of the analysis. Each finding is marked on a copy of the analyzed X-ray image and listed in a table. Additionally, the AI-Rad provides a “confidence score” (CS) on a scale of 1 (low) to 10 (high) for each finding, which expresses the algorithm´s certainty for the presence of that particular finding. The manufacturer has preset the AI-Rad only to report findings with a CS ≥ 6, whilst findings with a CS ≤ 5 are not displayed.

The AI-Rad (version VA23A) is designed to detect five specific radiographic findings: Pulmonary lesions, consolidation, atelectasis, pneumothorax and pleural effusion. Pulmonary lesions, as defined by the AI-Rad, include lung nodules (rounded or oval opacities < 3 cm in diameter) and lung masses (pulmonary, pleural or mediastinal lesions > 3 cm in diameter). To detect pneumothoraces, the AI-Rad screens for radiographic signs suggestive of air in the pleural space. Likewise, the AI-Rad screens for radiographic signs suggestive of fluid in the pleural space for the detection of pleural effusions. Atelectasis are defined as increased opacities accompanied by volume loss, which, in turn, can be an abnormal displacement of fissures, bronchi, vessels, the diaphragm, or the mediastinum. The AI-Rad defines consolidations as increased parenchymal attenuation. This definition includes homogeneous increases of parenchymal attenuation (consolidation) that obscures pulmonary vessels and bronchi as well as hazy increases of parenchymal attenuation (ground glass opacity) that do not obscure pulmonary vessels and bronchi.

### Reporting procedures and data collection

The report for each radiograph was written immediately after the examination. In most cases, the radiographs were evaluated in a consensus decision between a junior radiologist and a senior radiologist (> 20 years of experience). The radiologists were not aware of this study. Therefore, the written reports reflect the radiological routine without any external influencing factors. The evaluation of the radiographs by the AI-Rad was performed retrospectively.

The written reports were screened for the mentioning of the pre-defined radiographic findings (pulmonary lesions, consolidation, atelectasis, pneumothorax and pleural effusion). In case a certain pre-defined finding was not mentioned in the written report, it was considered as “not detected by the radiologist”. The findings detected by the AI-Rad were listed including the CS (confidence score).

### Ground truth

The ground truth for the data set was defined in a consensus decision by two radiologists (4 and 6 years of experience). In order to do so, further images (e.g. additional radiographs in lateral view, previous and/or follow-up X-ray examinations as well as CT scans) were taken into account.

While determining the ground truth, the overall image quality of the radiographs was rated on a 5-point Likert scale (1 = very poor image quality, 5 = excellent image quality). In addition, the reason for a potentially suboptimal image quality was determined.

### Statistical analysis

Data processing and descriptive statistical analyses as well as graphical illustration were performed using the statistical software R and RStudio (R Core Team (2021). R: A language and environment for statistical computing. R Foundation for Statistical Computing, Vienna, Austria. RStudio Version 1.4.1106). For the written report as well as the AI-Rad analysis, the sensitivity, specificity, positive (PPV) and negative predictive value (NPV) as well as the false discovery rate (FDR) and the false omission rate (FOR) were calculated for the detection of each pre-defined finding. Furthermore, receiver operating characteristic (ROC) curves were created and the area under the curve (AUC) was calculated to illustrate the performances.

### Ethical approval

Institutional Review Board Statement: The study was conducted according to the guidelines of the Declaration of Helsinki and approved by the Ethics Committee of the Faculty of Medicine of the Ruhr-University Bochum.

### Informed consent

Patient consent was waived by the Ethics Committee of the Faculty of Medicine of the Ruhr-University Bochum due to the retrospective study design.

## Results

Chest radiographs of 499 patients were analyzed in the present study. The mean age was 65.4 ± 17.0 (median 67.6, range 22–97) years.

Overall, the image quality of the great majority of radiographs was “good” or “excellent”. Only 1.2% of the radiographs were rated “appropriate”. The most frequently cited reason for suboptimal image quality was “overlapping soft tissue”. Details on the image quality are summarized in Table 1.

**Table 1:** Rating of the image quality and reasons for suboptimal image quality.

	Overall (n = 499)
Image quality	
(1) Very poor	0 (0%)
(2) Poor	0 (0%)
(3) Appropriate	6 (1.2%)
(4) Good	277 (55.5%)
(5) Excellent	216 (43.3%)
Reason for suboptimal	
Rotated	15 (3.0%)
Image quality	
Overlapping soft tissue	222 (44.5%)
Insufficient inspiration	10 (2.0%)
Lung not fully displayed	36 (7.2%)

### Ground truth

Overall, 499 X-ray examinations were included in the present study of which 386 examinations included radiographs in p.a. and lateral view, 113 examinations consisted of radiography solely in p.a. view.

To determine the ground truth not only the particularly in this study included X-ray images were evaluated, but also additional examinations were considered. In 375 of the 499 included cases, additional X-ray examinations and/or CT scans were available at the time, when the ground truth was defined.

In terms of additional radiographs, 332 patients had at least one additional X-ray examination of the chest. In 299 cases, previously acquired X-ray images were available. In 136 cases, follow-up X-ray images were available. In 103 cases, both previous as well as follow-up X-ray images were available.

Likewise, 237 patients had at least one CT examination that included the chest. In 186 cases, a CT scan that was acquired before the date of the in this study included radiograph was available. In 121 cases, a CT scan that was acquired after the date of the in this study included radiograph was available. In 70 cases, CT scans that were acquired before as well as after the date of the in this study included radiograph were available.

On 312 of the 499 analyzed (62.5%) radiographs, none of the pre-defined findings was detected. Accordingly, on 187 radiographs (37.4%) at least one of the pre-defined findings was detected; out of these radiographs, the majority had one (n = 99) or two (n = 62) pre-defined findings. Table 2 shows the distribution of the pre-defined findings.

**Table 2:** Total number of verified findings on all analyzed radiographs.

	Overall (n = 499)
Lung lesion	
Yes	58 (11.6%)
No	441 (88.4%)
Consolidation	
Yes	51 (10.2%)
No	448 (89.8%)
Atelectasis	
Yes	90 (18.0%)
No	409 (82.0%)
Pleural effusion	
Yes	97 (19.4%)
No	402 (80.6%)
Pneumothorax	
Yes	10 (2.0%)
No	489 (98.0%)

The written report and the AI-Rad analysis came to the same result in 251 cases (50.3%) and disagreed in 248 cases (49.7%). In 366 cases (73.3%), the written report agreed with the ground truth and in 133 cases (26.7%), the written report disagreed with the ground truth. Likewise, in 276 cases (55.3%), the AI-Rad agreed with the ground truth and in 223 cases (44.7%), the AI-Rad disagreed with the ground truth.

### Lung lesions

**Figure 1:**
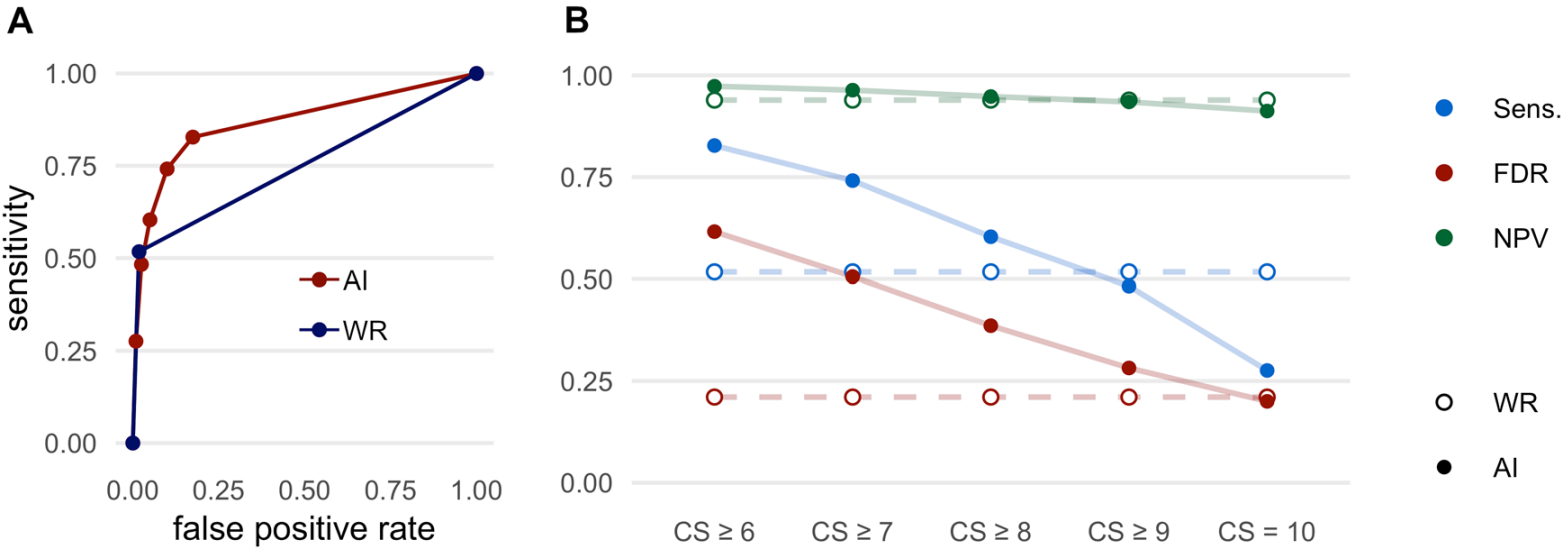
Performance metrics for the detection of lung lesions. (**A**) Receiver operating characteristic (ROC) curves displaying the performance of the AI Rad Companion Chest X-ray (AI) and the written report (WR) for the detection of lung lesions (area under the curve (AUC) AI = 0.867/WR = 0.750). (**B**) Sensitivity (Sens.), negative predictive value (NPV) and false discovery rate (FDR) of the AI Rad Companion Chest X-ray (AI) and the radiologists (WR = written report) for the detection of lung lesions. The sensitivity of the AI is considerably higher at a (confidence score =) CS ≥ 6; at the same time, the FDR is considerably higher. At CS = 10, the FDR is comparable to the WR, but the sensitivity is considerably lower. The NPV of the AI is high at all CS.

**Figure 2:**
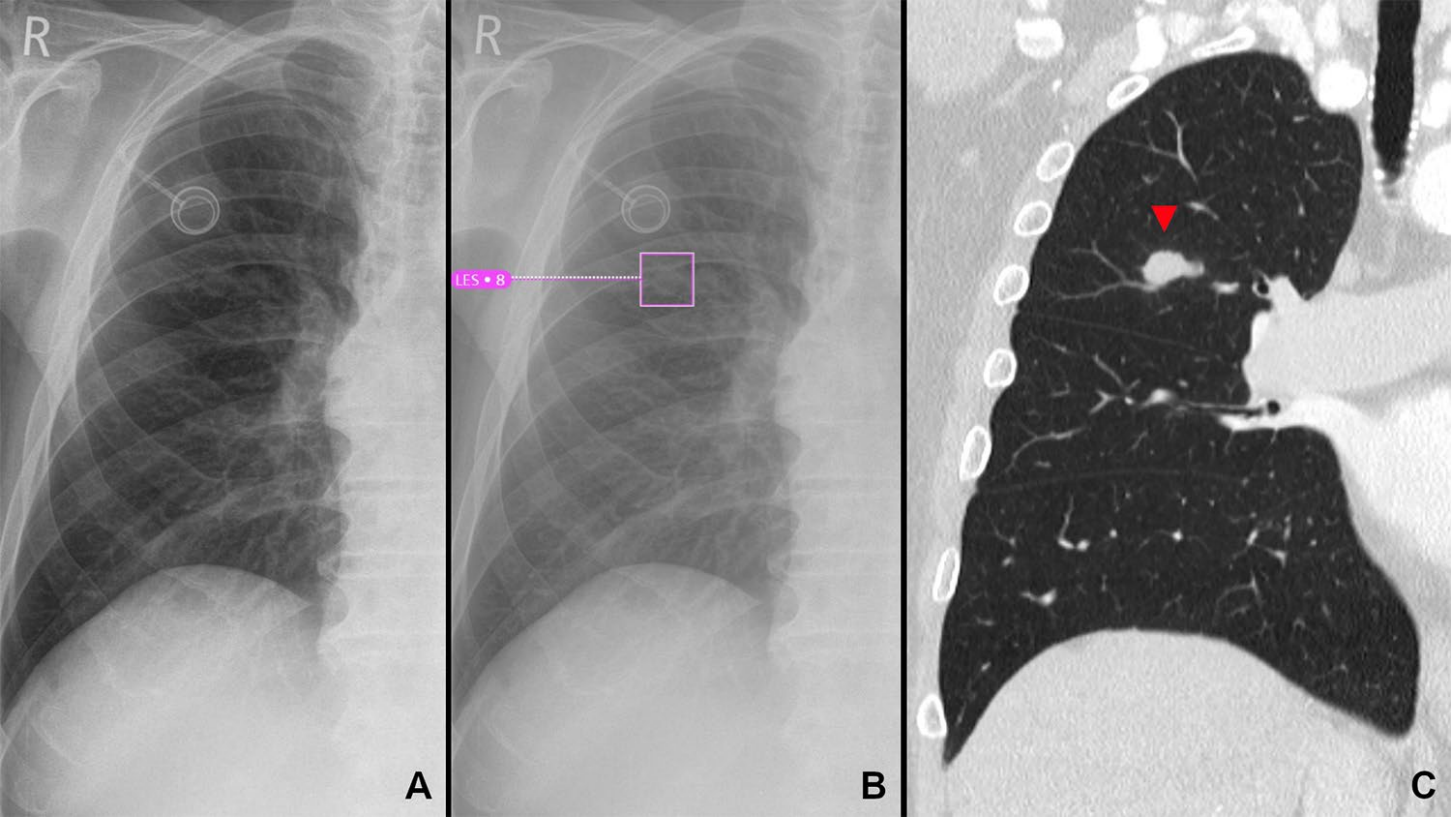
Example of a lung lesion detected by the AI Rad Companion Chest X-ray (**A**, **B**) that was confirmed by a CT scan of the chest (**C**): 74 year old, male patient diagnosed with a carcinoma of the tongue 2 years ago.

The results regarding the detection of lung lesions are shown in Fig. 1 and Table 3. An example for the detection of a lung lesion is shown in Fig. 2. Considering all CS (CS ≥ 6), the AI-Rad offered high sensitivity (0.83) and specificity (0.83) for the detection of lung lesions with an excellent NPV (0.97), but high FDR (0.62). With increasing level of CS, the FDR (0.20 at CS = 10) decreased markedly. At the same time, however, the sensitivity (0.28 at CS = 10) decreased markedly. The NPV remained high (0.91 at CS = 10).

**Table 3:** Performance metrics of the radiologists (WR = written report) and the AI Rad Companion Chest X-ray (AI-Rad) for the detection of lung lesions.

	Sens	Spec	PPV	FDR	NPV	FOR
WR	0.52	0.98	0.79	0.21	0.94	0.06
AI-Rad CS ≥ 6	0.83	0.83	0.38	0.62	0.97	0.03
AI-Rad CS ≥ 7	0.74	0.90	0.49	0.51	0.96	0.04
AI-Rad CS ≥ 8	0.60	0.95	0.61	0.39	0.95	0.05
AI-Rad CS ≥ 9	0.48	0.98	0.72	0.28	0.93	0.07
AI-Rad CS = 10	0.28	0.99	0.80	0.20	0.91	0.09

*CS* confidence score, *PPV* positive predictive value, *NPV* negative predictive value, *FDR* false discovery rate, *FOR* false omission rate.

The sensitivity of the written report for the detection of lung lesions was comparatively low (0.52). The specificity (0.98) as well as the NPV (0.94) were excellent. At the same time, the FDR was comparatively low (0.21).

### Consolidation

**Figure 3:**
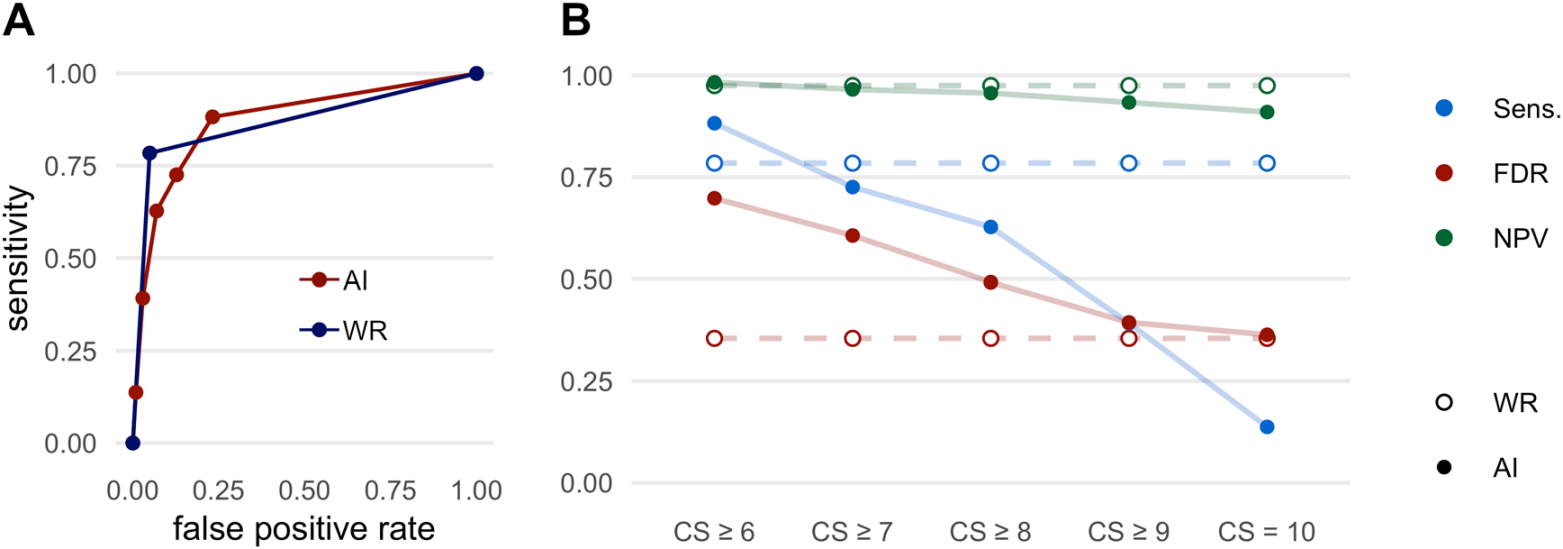
Performance metrics for the detection of consolidations. (**A**) Receiver operating characteristic (ROC) curves displaying the performance of the AI Rad Companion Chest X-ray (AI) and the radiologists (WR = written report) for the detection of consolidations (area under the curve (AUC) AI = 0.873/WR = 0.868). (**B**) Sensitivity (Sens.), negative predictive value (NPV) and false discovery rate (FDR) of the AI Rad Companion Chest X-ray (AI) and the radiologists (WR = written report) for the detection of consolidations. The sensitivity of the AI is slightly higher at a (confidence score =) CS ≥ 6; at the same time, the FDR is markedly higher. At CS = 10, the FDR is comparable to the WR, but the sensitivity is much lower. The NPV of the AI is high at all CS.

**Figure 4:**
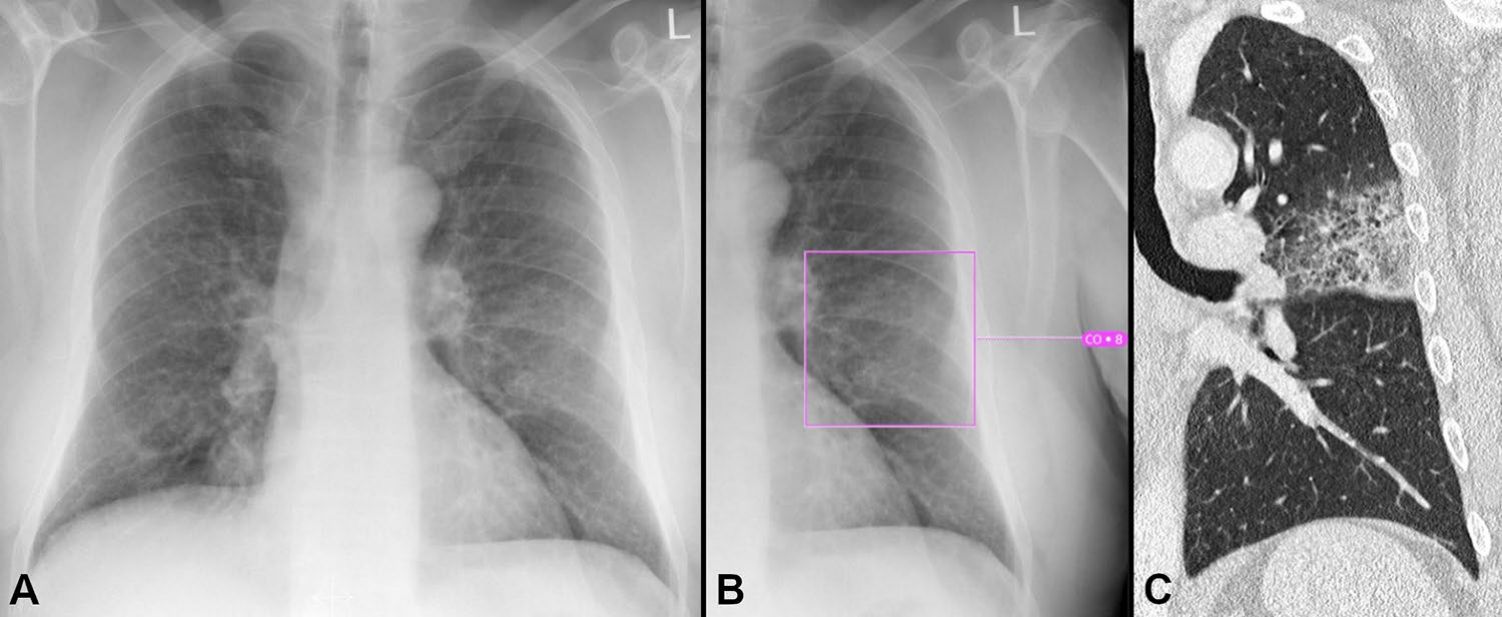
Example of a consolidation detected by the AI Rad Companion Chest X-ray (**A**, **B**) that was confirmed by a CT scan (**C**): 72 year old, male patient admitted to the hospital with fever, dyspnea and severe cough.

The results regarding the detection of consolidations are shown in Fig. 3 and Table 4. An example for the detection of consolidations is shown in Fig. 4. The AI-Rad offers good sensitivity (0.88) and specificity (0.77) for the detection of consolidations, when considering all CS (CS ≥ 6). With increasing CS, the sensitivity decreases markedly (0.14 at CS = 10), whereas the specificity increases (0.99 at CS = 10). The NPV is excellent at all CS (0.98 at CS ≥ 6; 0.91 at CS = 10). The FDR is relatively high (0.70 at CS ≥ 6), when considering all CS, but decreases noticeably with increasing CS (0.36 at CS = 10).

**Table 4:** Performance metrics of the radiologists (WR = written report) and the AI Rad Companion Chest X-ray (AI-Rad) for the detection of consolidations.

	Sens	Spec	PPV	FDR	NPV	FOR
WR	0.78	0.95	0.65	0.35	0.97	0.03
AI-Rad CS ≥ 6	0.88	0.77	0.30	0.70	0.98	0.02
AI-Rad CS ≥ 7	0.73	0.87	0.39	0.61	0.97	0.03
AI-Rad CS ≥ 8	0.63	0.93	0.51	0.49	0.96	0.04
AI-Rad CS ≥ 9	0.39	0.97	0.61	0.39	0.93	0.07
AI-Rad CS = 10	0.14	0.99	0.64	0.36	0.91	0.09

*CS* confidence score, *PPV* positive predictive value, *NPV* negative predictive value, *FDR* false discovery rate, *FOR* false omission rate.

The sensitivity of the written report for the detection of consolidations was good (0.78). The specificity (0.98) as well as the NPV (0.94) were excellent. In addition, the FDR was comparatively low (0.35).

### Atelectasis

**Figure 5:**
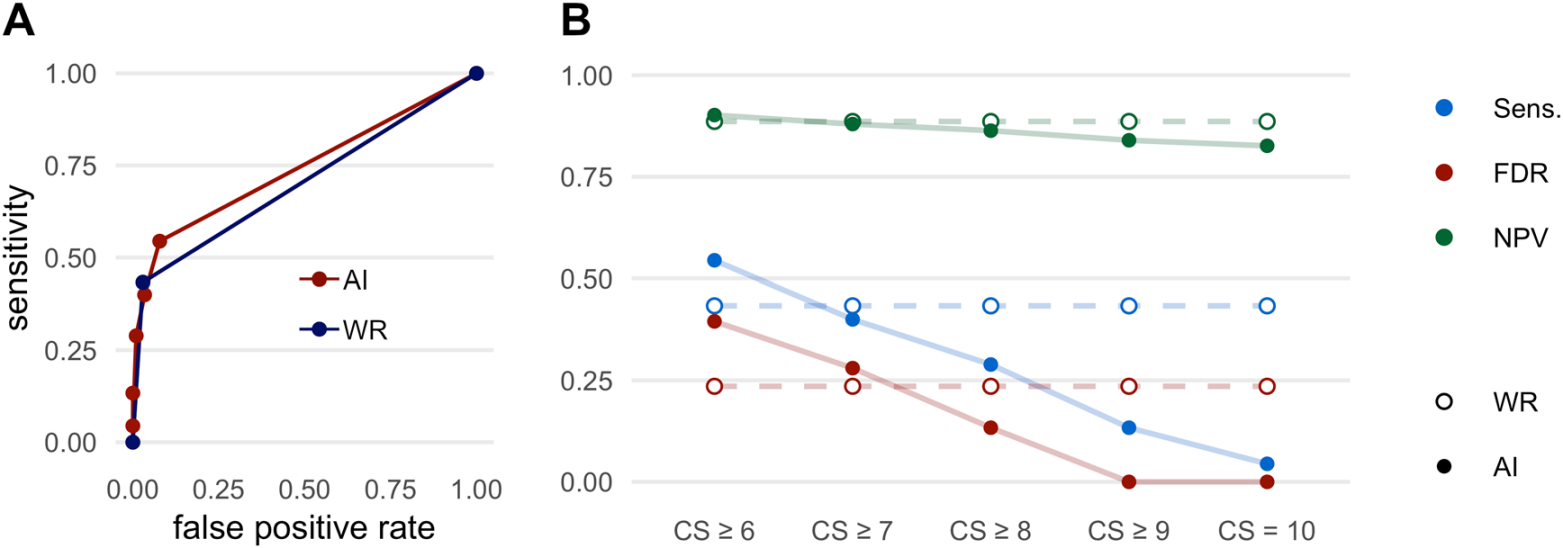
Performance metrics for the detection of atelectasis. (**A**) Receiver operating characteristic (ROC) curves displaying the performance of the AI Rad Companion Chest X-ray (AI) and the radiologists (WR = written report) for the detection of atelectasis (area under the curve (AUC) AI = 0.743/WR = 0.702). (**B**) Sensitivity (Sens.), negative predictive value (NPV) and false discovery rate (FDR) of the AI Rad Companion Chest X-ray (AI) and the radiologists (WR = written report) for the detection of atelectasis. The sensitivity of the AI is slightly higher at a (confidence score =) CS ≥ 6; at the same time, the FDR is slightly higher. At CS ≥ 7, sensitivity and FDR of the AI and the WR are on a similar level. At CS ≥ 8, the sensitivity as well as the FDR decrease markedly. The NPV of the AI is high at all CS.

The results regarding the detection of atelectasis are shown in Fig. 5 and Table 5. The AI-Rad offers moderate sensitivity (0.54 at CS ≥ 6) for the detection of atelectasis that decreases markedly with increasing CS (0.04 at CS = 10). The specificity (0.92 at CS ≥ 6) as well as the NPV (0.90 at CS ≥ 6) remain very high at all CS. The FDR is highest when considering all CS (0.40 at CS ≥ 6) and decreases markedly with increasing level of CS.

**Table 5:** Performance metrics of the radiologists (WR = written report) and the AI Rad Companion Chest X-ray (AI-Rad) for the detection of atelectasis.

	Sens	Spec	PPV	FDR	NPV	FOR
WR	0.43	0.97	0.76	0.24	0.89	0.11
AI-Rad CS ≥ 6	0.54	0.92	0.60	0.40	0.90	0.10
AI-Rad CS ≥ 7	0.40	0.97	0.72	0.28	0.88	0.12
AI-Rad CS ≥ 8	0.29	0.99	0.87	0.13	0.86	0.14
AI-Rad CS ≥ 9	0.13	1.00	1.00	0.00	0.84	0.16
AI-Rad CS = 10	0.04	1.00	1.00	0.00	0.83	0.17

*CS* confidence score, *PPV* positive predictive value, *NPV* negative predictive value, *FDR* false discovery rate, *FOR* false omission rate.

Likewise, the written report offers moderate sensitivity (0.43) for the detection of atelectasis. The specificity (0.97) as well as the NPV (0.89) are excellent. The FDR is on a low level (0.24).

### Pneumothorax

**Figure 6:**
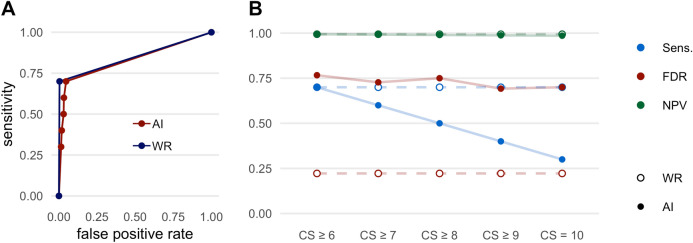
Performance metrics for the detection of pneumothoraces. (**A**) Receiver operating characteristic (ROC) curves displaying the performance of the AI Rad Companion Chest X-ray (AI) and the radiologists (WR = written report) for the detection of pneumothoraces (area under the curve (AUC) AI = 0.830/WR = 0.848). (**B**) Sensitivity (Sens.), negative predictive value (NPV) and false discovery rate (FDR) of the AI Rad Companion Chest X-ray (AI) and the radiologists (WR = written report) for the detection of pneumothoraces. CS = confidence score.

The results regarding the detection of pneumothoraces are shown in Fig. 6 and Table 6. When analyzing the performance metrics for the detection of pneumothoraces, it must be noted that the prevalence of pneumothoraces was considerably low in the cohort (2.0%), which influences the overall calculation of the performance metrics.

**Table 6:** Performance metrics of the radiologists (WR = written report) and the AI Rad Companion Chest X-ray (AI-Rad) for the detection of pneumothoraces.

	Sens	Spec	PPV	FDR	NPV	FOR
WR	0.70	1.00	0.78	0.22	0.99	0.01
AI-Rad CS ≥ 6	0.70	0.95	0.23	0.77	0.99	0.01
AI-Rad CS ≥ 7	0.60	0.97	0.27	0.73	0.99	0.01
AI-Rad CS ≥ 8	0.50	0.97	0.25	0.75	0.99	0.01
AI-Rad CS ≥ 9	0.40	0.98	0.31	0.69	0.99	0.01
AI-Rad CS = 10	0.30	0.99	0.30	0.70	0.99	0.01

*CS* confidence score, *PPV* positive predictive value, *NPV* negative predictive value, *FDR* false discovery rate, *FOR* false omission rate.

**Figure 7:**
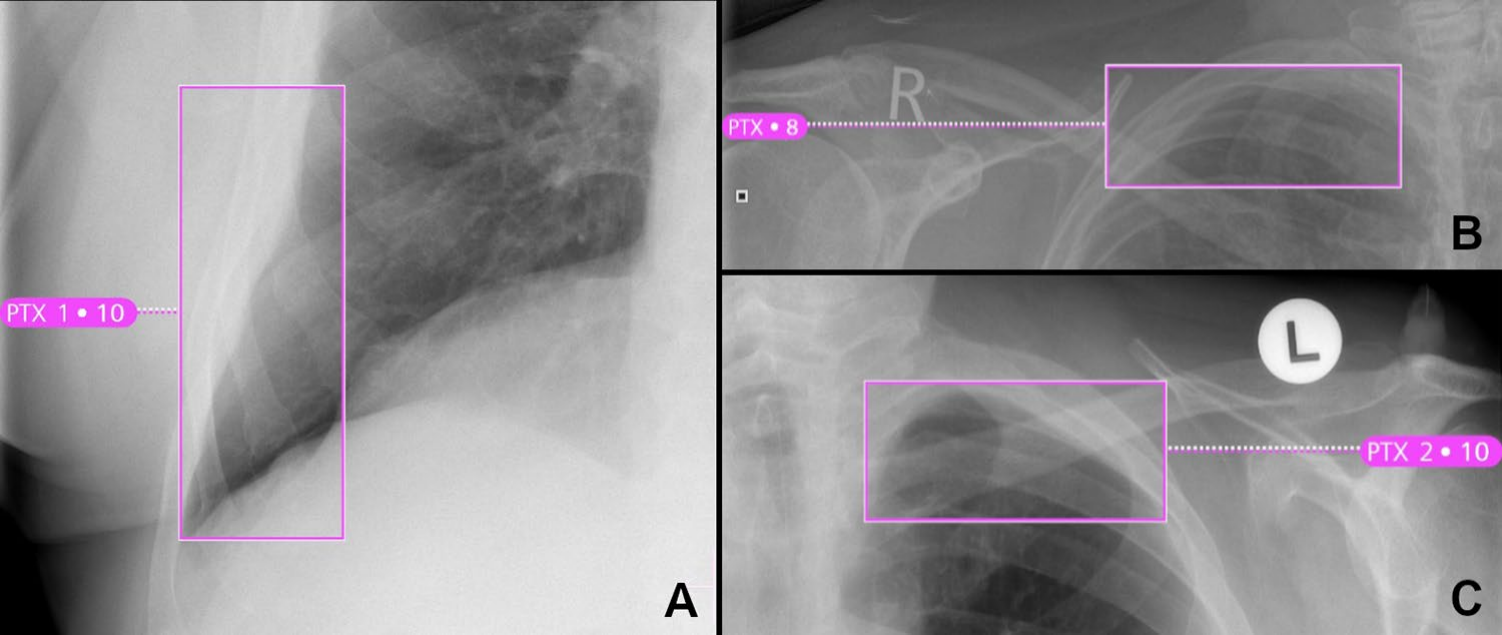
Three examples of false positively indicated pneumothoraces (A-C) by the AI Rad Companion Chest X-ray.

The AI-Rad offers a good sensitivity for the detection of pneumothoraces when considering all levels of CS (0.70 at CS ≥ 6). However, the sensitivity decreases markedly with increasing level of CS (0.30 at CS = 10). Both specificity as well as NPV are excellent at all CS. The FDR is comparatively high at all levels of CS (0.70 at CS = 10). Described in absolute numbers; the AI-Rad detected 7 out of 10 pneumothoraces correctly. At the same time, the AI-Rad indicated 23 pneumothoraces false positively (see also Fig. 7).

The written report offers good sensitivity for the detection of pneumothoraces (0.70). The specificity (1.0), the NPV (0.99) as well as the FDR (0.22) are excellent.

### Pleural effusion

**Figure 8:**
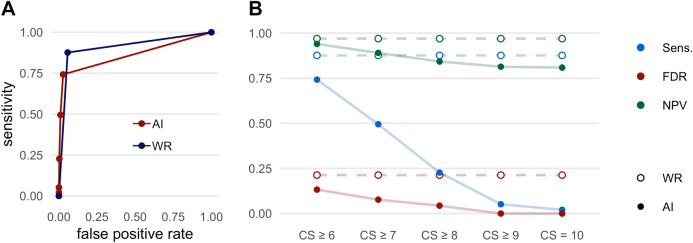
Performance metrics for the detection of pleural effusions. (**A**) Receiver operating characteristic (ROC) curves displaying the performance of the AI Rad Companion Chest X-ray (AI) and the radiologists (WR = written report) for the detection of pleural effusions (area under the curve (AUC) AI = 0.861 / WR = 0.910). (**B**) Sensitivity (Sens.), negative predictive value (NPV) and false discovery rate (FDR) of the AI Rad Companion Chest X-ray (AI) and the radiologists (WR = written report) for the detection of pleural effusions. The sensitivity of the AI is inferior to the sensitivity of the WR at all CS (= confidence scores).

The results regarding the detection of pleural effusions are shown in Fig. 8 and Table 7. The AI-Rad offers good sensitivity for detecting pleural effusions when considering all levels of CS (0.74 at CS ≥ 6). However, the sensitivity decreases dramatically with increasing level of CS (0.02 at CS = 10). The specificity was excellent at all levels of CS. The NPV decreased slightly with increasing level of CS, but remained on a very good level (e.g. 0.81 at CS = 10). The FDR was very low at all levels of CS (e.g. 0.13 at CS ≥ 6).

**Table 7:** Performance metrics of the radiologists (WR = written report) and the AI Rad Companion Chest X-ray (AI-Rad) for the detection of pleural effusions.

	Sens	Spec	PPV	FDR	NPV	FOR
WR	0.88	0.94	0.79	0.21	0.97	0.03
AI-Rad CS ≥ 6	0.74	0.97	0.87	0.13	0.94	0.06
AI-Rad CS ≥ 7	0.49	0.99	0.92	0.08	0.89	0.11
AI-Rad CS ≥ 8	0.23	1.00	0.96	0.04	0.84	0.16
AI-Rad CS ≥ 9	0.05	1.00	1.00	0.00	0.81	0.19
AI-Rad CS = 10	0.02	1.00	1.00	0.00	0.81	0.19

*CS* confidence score, *PPV* positive predictive value, *NPV* negative predictive value, *FDR* false discovery rate, *FOR* false omission rate.

The written report offered very good sensitivity (0.88) and excellent specificity (0.94) as well as NPV (0.97) for the detection of pleural effusions. At the same time, the FDR was low (0.21).

## Discussion

The purpose of the present study was to evaluate the performance of the AI-Rad (version VA23A) by analyzing the performance metrics of the AI-Rad and clinically working radiologists. The findings described in the written reports and the findings detected by the AI-Rad were compared to the findings of a ground truth reading, which was accomplished by a consensus agreement of two radiologists after evaluating additional radiographs (e.g. lateral view) and CT examinations (if available).

For the interpretation of the performance metrics of the AI-Rad, it is important to consider the different CS that are provided for each detected finding. The CS expresses the algorithm´s certainty for the presence of that particular finding. The AI-Rad might offer higher sensitivity for certain findings compared to the written report when considering the lowest CS (≥ 6). However, at the same time, the FDR of the AI-Rad at this CS might also be considerably higher. Likewise, at a higher CS, the AI-Rad might offer a similar FDR compared to the written report, but with a considerably lower sensitivity. Therefore, the different CS are important when evaluating the reported findings of the AI-Rad.

The sensitivity of the AI-Rad for the detection of lung lesions was superior in comparison to the sensitivity of the written report (0.83 (AI-Rad at CS ≥ 6) versus 0.52 (WR)). However, it has to be noted that, unlike the AI-Rad, radiologists immediately evaluate the findings they detect and decide whether it is worth mentioning in the written report. It is conceivable that a small, calcified granuloma, for example, that has been present for a long time may not be mentioned in the written report, but is indicated by the AI-Rad.

Furthermore, the sensitivity of the written report for the detection of lung lesions in the present study is comparable to previously published data. Homayounieh et al., for example, report on a mean sensitivity of 45% among nine radiologists with different levels of experience for the detection of pulmonary nodules^15^. The sensitivity of the AI-Rad in the present study is also comparable to previous published data. Yoo et al., for example, report on an artificial intelligence algorithm for lung nodule detection and describe a sensitivity of 86%^18^.

The superior sensitivity of the AI-Rad for the detection of lung lesions (at CS ≥ 6), however, is accompanied by a markedly higher FDR compared to the written report (0.62 (AI-Rad at CS ≥ 6) versus 0.21 (WR)). Indeed, the AI-Rad wrongly indicated ECG electrodes or the nipple as lung lesions in several cases. Calcifications of the costal cartilage are also often misinterpreted by the AI-Rad. Consequently, radiologists need to check each indicated finding with a CS ≥ 6 as the number of false positive findings is considerably high. When the AI-Rad reports a lung lesion with the CS = 10, it is more likely to be a true positive finding as the FDR is markedly lower (0.20 compared to 0.62 at CS ≥ 6).

In terms of detecting lung lesions, a benefit of the AI-Rad for clinical radiologists may be the high NPV (0.91–0.97; depending on the CS), which is comparable to the NPV of the written report (0.97). When taking the evaluation of the AI-Rad into account, radiologists may re-insure their own negative search for lung lesions, which may increase their confidence in their report.

In terms of detecting consolidations, the AI-Rad offers slightly higher sensitivity (0.88 (AI-Rad at CS ≥ 6) versus 0.78 (WR)) compared to the written report. However, the higher sensitivity is accompanied by a higher FDR (0.70 (AI-Rad at CS ≥ 6) versus 0.35 (WR)). Therefore, radiologists might benefit from the higher sensitivity, but need to re-evaluate the indicated findings of the AI-Rad carefully. At CS = 10, the FDR of the AI-Rad is comparable to the WR (0.36 (AI-Rad at CS = 10) versus 0.35 (WR)), but the sensitivity of the AI-Rad decreased markedly (0.14 (AI-Rad at CS = 10) versus 0.78 (WR)).

These performance metrics of the AI-Rad regarding the detection of consolidations are in line with previously published data. Rueckel et al., for example, report on minor differences in the performance of an AI algorithm and board-certified radiologists for the detection of pneumonia on chest radiographs^19^. In addition, Yee et al. report on a comparable sensitivity (84.1%) of their neural network for the detection of pneumonia on chest radiographs^20^.

In terms of detecting consolidations, the high NPV (0.91–0.98; depending of the CS) of the AI-Rad may be a benefit for radiologists in clinical practice as they can reliably re-insure their own negative search for consolidations.

Similar to the detection of consolidations, the AI-Rad can provide slightly higher sensitivity for the detection of atelectasis compared to the written report (0.54 (AI-Rad at CS ≥ 6) versus 0.43 (WR)). However, it has to be noted that—similar to the arguments mentioned for the detection of lung lesions—it remains unclear whether small atelectasis have been detected by the radiologists, but were not considered worth mentioning in the written report. At CS ≥ 6, the FDR of the AI-Rad is higher compared to the written report (0.40 (AI-Rad at CS ≥ 6) versus 0.24 (WR)). Sensitivity as well as FDR decrease markedly with increasing CS. The NPV of the AI-Rad and the written report for the detection of atelectasis are on a high level (0.83–0.90 (AI-Rad; depending of the CS) versus 0.89 (WR)).

Compared to the AI-Rad, the written report achieved higher sensitivity for the detection of pleural effusions (0.74 (AI-Rad at CS ≥ 6) versus 0.88 (WR)). This might be accounted to the additional lateral view radiographs that are not taken into account by the AI-Rad, but are helpful in detecting smaller pleural effusions. The NPV (0.94 (AI-Rad at CS ≥ 6) versus 0.97 (WR)) of the AI-Rad and the written report for the detection of pleural effusions are comparable. This is in line with an earlier study conducted by Rueckel et al., who found only minor differences in the performance of an AI algorithm and board-certified radiologists for the detection of pleural effusions on chest radiographs^19^.

The performance metrics regarding the detection of pneumothoraces calculated in the present study are most likely not representative due to the low prevalence of pneumothoraces in our cohort (2.0%). However, during the systematic analysis of the radiographs for establishing the ground truth, we noticed that the AI-Rad indicates a considerably high number of pneumothoraces that are false positive. Therefore, according to our experience, it is conceivable that the FDR would be comparatively high even with a higher prevalence in the cohort. Nevertheless, future studies with higher prevalence need to evaluate reliably the performance of the AI-Rad for the detection of pneumothoraces.

The present study has certain limitations: (1) The AI-Rad is intended to be a supporting tool whose output is considered by radiologists before making their final decision while writing reports. However, the present study evaluated the performance of the AI-Rad alone and compared it to the performance of radiologists in the clinical routine without the assistance of an AI application. (2) As previously published studies show, less experienced radiologists are more likely to benefit from the support of an AI application^15^. However, the present study aimed to compare the overall performance of radiologists in the clinical routine and therefore did not differentiate between the individual experience of each radiologist. (3) The analysis in the present study focused on the list of findings provided by the AI-Rad, rather than the location of a finding indicated by the AI-Rad. Therefore, it is possible that the AI-Rad may have correctly listed a finding on the report sheet, but indicates it in the wrong location. (4) Unlike the AI-Rad, radiologists are able to consider lateral view radiographs and previously conducted radiographs for comparison. (5) As explained above, the conclusions regarding the performance of the AI-Rad for the detection of pneumothoraces are limited because of the low prevalence of pneumothoraces in this cohort. (6) The overall image quality of the chest radiographs was very good. The performance of the AI-Rad regarding chest radiographs with poor image quality was not evaluated in the present study.

## Conclusions

The results of the present study indicate that the AI-Rad can offer a slightly higher sensitivity for the detection of certain findings (lung lesions, consolidations and atelectasis) compared to the written report. However, this advantage is partially offset by the disadvantage of a higher FDR of the AI-Rad. Consequently, radiologist need to carefully re-evaluate and verify each finding indicated by the AI-Rad.

At the current stage of development, it is conceivable that the high NPVs for the detection of the pre-defined findings are the greatest benefit of the AI-Rad. Radiologists re-insuring their own negative search for pathologies in a chest radiograph by considering the evaluation of the AI-Rad may have higher diagnostic confidence in their reports leading to faster reporting.

## Data Availability

The data are available from the corresponding author on reasonable request.
